# Supplementary material for: Comparative Genomics Reveal Phylogenetic Relationship and Chromosomal Evolutionary Events of Eight Cervidae Species
Source: Animals (Basel). 2024 Mar 30;14(7):1063. doi: 10.3390/ani14071063 (PMC11010878; doi:10.3390/ani14071063)
Supplement: Supplementary file 1 [file animals-14-01063-s001.zip › Figure S1 Phylogenetic tree with bootstrap value .pdf]

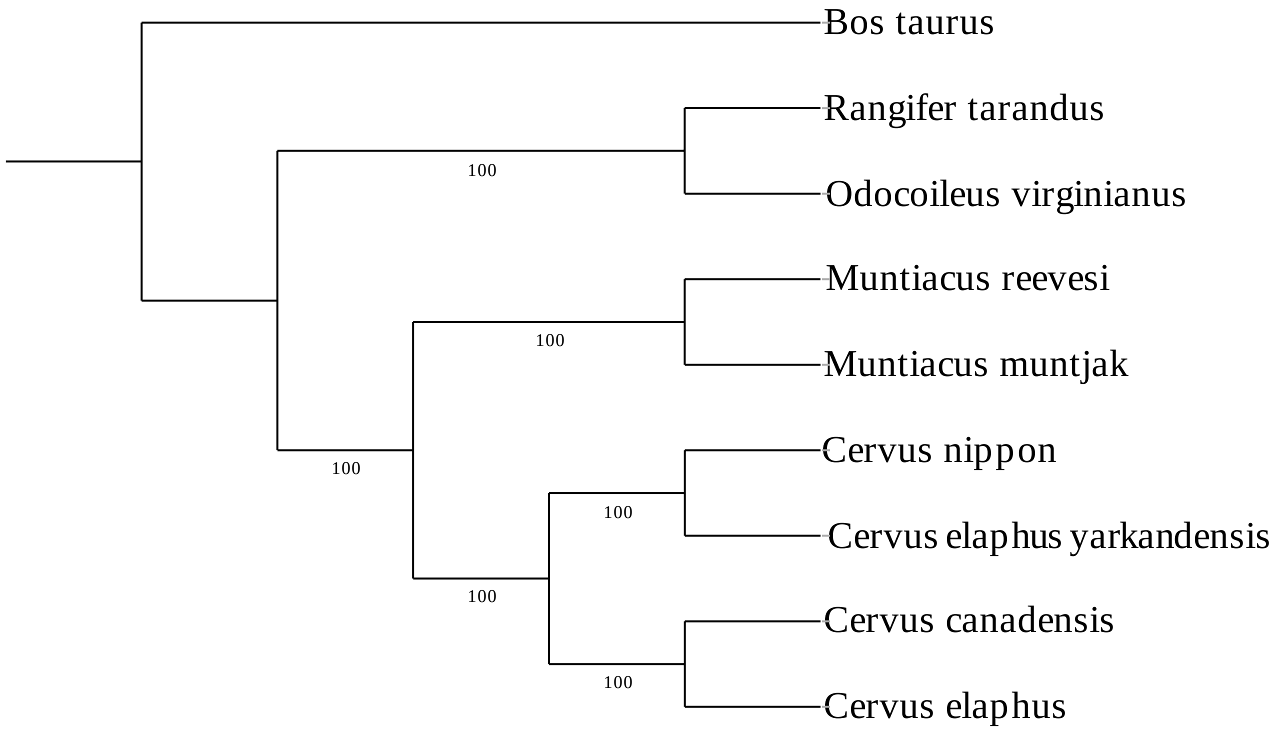

Figure S1. Phylogenetic tree with bootstrap value

The bootstrap value for each clade was 100. The structure of the clade was obtained 1000 times out of 1000 sample reconstructions of the data.
