# Supplementary figures and images for: Comparative Genomics Reveal Phylogenetic Relationship and Chromosomal Evolutionary Events of Eight Cervidae Species
Source: Animals (Basel). 2024 Mar 30;14(7):1063. doi: 10.3390/ani14071063 (PMC11010878; doi:10.3390/ani14071063)

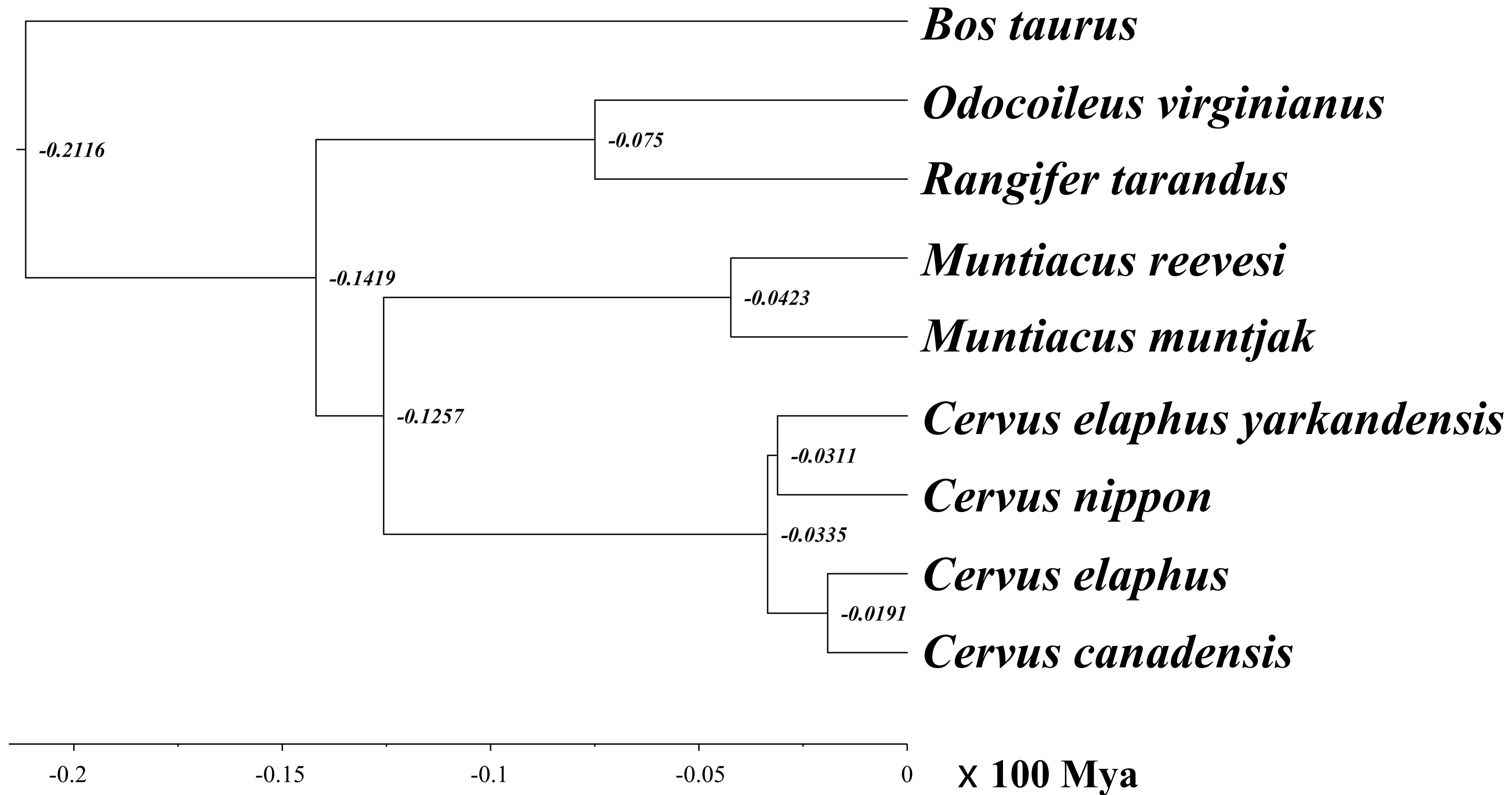

Figure S2. Phylogenetic tree with divergence time

Supplement: Supplementary file 1 [file animals-14-01063-s001.zip › Figure S2 Phylogenetic tree with divergence time.pdf]
